# Silver and Copper Nanoparticles as Non‐Antibiotic Therapy for Postpartum Endometritis in Dairy Cows

**DOI:** 10.1002/vms3.71113

**Published:** 2026-08-05

**Authors:** Meysam Makki, Seyed Mohammad Taha Mousavi Shoushtari, Saad Gooraninejad, Mohammad Khosravi

**Affiliations:** ^1^ Department of Clinical Sciences Faculty of Veterinary Medicine Shahid Chamran University of Ahvaz Ahvaz Iran; ^2^ Faculty of Veterinary Medicine Shahid Chamran University of Ahvaz Ahvaz Iran; ^3^ Department of Pathobiology Faculty of Veterinary Medicine Shahid Chamran University of Ahvaz Ahvaz Iran

**Keywords:** clinical endometritis, copper, dairy cows, nanoparticles, non‐antibiotic therapy, silver

## Abstract

**Objectives:**

Postpartum clinical endometritis reduces reproductive efficiency in dairy cows. Increasing antimicrobial resistance has created an urgent need for non‐antibiotic therapeutic alternatives. This study evaluated the efficacy of intrauterine silver and copper nanoparticles compared with prostaglandin (PGF2α) and 50% dextrose in the treatment of clinical endometritis.

**Methods:**

A total of 210 cows were screened 30 ± 3 days postpartum, and 60 cows diagnosed with clinical endometritis were randomly assigned to four treatment groups: 50% dextrose (*n* = 15), PGF2α (*n* = 15), silver and copper nanoparticles (*n* = 15), and untreated controls (*n* = 15). Clinical and cytological evaluations were performed 14 days after treatment.

**Results:**

The clinical cure rates were 80% for both the dextrose and nanoparticle groups, 66.7% for the PGF2α group, and 46.7% for the control group. The mean percentage of neutrophils was significantly lower (*p* < 0.05) in cows treated with dextrose or nanoparticles compared with the control group. Reproductive indices, including the number of inseminations and days open, were improved in the treated groups.

**Conclusion:**

These findings indicate that silver and copper nanoparticles exhibit therapeutic efficacy comparable to hypertonic dextrose and superior to prostaglandin in resolving postpartum clinical endometritis. The results support the potential use of metal nanoparticles as a non‐antibiotic, residue‐free alternative for improving uterine health and fertility in dairy herds.

## Graphical abstract

Postpartum dairy cows with clinical endometritis (purulent or mucopurulent vaginal discharge and >10% polymorphonuclear neutrophils [PMNs]) received intrauterine copper–silver nanoparticles, PGF2α, or no treatment. Clinical and cytological responses were assessed 14 days later to evaluate the efficacy of copper–silver nanoparticles as a non‐antibiotic therapeutic strategy.

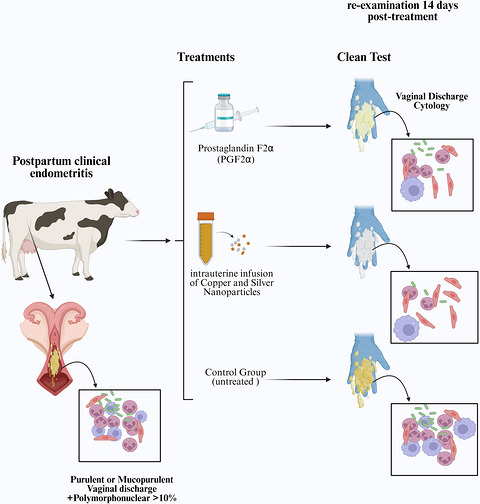

## 1 Introduction

Clinical endometritis (CE) is one of the most prevalent uterine disorders affecting reproductive performance in dairy cows. Several potential risk factors, including body condition score (BCS), ovarian status, calving status, parity, and placental expulsion, were evaluated for their association with clinical endometritis in dairy cows (Table 1) (Vallejo‐Timaran et al. 2021). It is characterized by the presence of mucopurulent or purulent uterine discharge beyond 21 days postpartum and is associated with prolonged days open, reduced conception rates, and economic losses to dairy producers (Sheldon et al. 2006). Postpartum endometritis is commonly associated with opportunistic bacterial pathogens that colonize the uterus during or shortly after parturition, particularly *Trueperella pyogenes*, *Escherichia coli*, *Fusobacterium necrophorum*, and *Prevotella* spp. Conventional treatments for CE mainly rely on antibiotics or prostaglandin F2α (PGF2α) (Ahmadi et al. 2019); however, increasing antimicrobial resistance and concerns regarding drug residues in milk and meat have prompted growing interest in non‐antibiotic alternatives. Therefore, increasing attention has been directed toward the use of non‐antimicrobial agents and biological preparations for the treatment of uterine infections in dairy cows. Several non‐antibiotic treatment strategies with minimal public health concerns have been investigated, including intrauterine infusion of blood serum (Aghamiri et al. 2022), platelet‐rich plasma (Marini et al. 2016), hypertonic solution of 50% dextrose (Brick et al. 2012), liquid paraffin (Ahmadi et al. 2019), and proteolytic enzymes (Singh et al. 2020). Among emerging alternatives, metallic nanoparticles (NPs) have attracted increasing attention because of their broad‐spectrum antimicrobial activity and low risk of inducing bacterial resistance. In recent years, metallic NPs such as silver (Ag NPs) and copper (Cu NPs) have demonstrated potent antimicrobial activity against a wide range of pathogens, offering potential therapeutic value in veterinary medicine (Chatterjee et al. 2014). Silver NPs possess strong antibacterial properties and can penetrate bacterial cell walls, alter membrane structure, increase membrane permeability, generate reactive oxygen species, and interfere with DNA replication through the release of silver ions (Makki et al. 2017; Yin et al. 2020). Copper NPs have also demonstrated significant bactericidal activity against a variety of bacterial pathogens (Chatterjee et al. 2014). Cu NPs were found to cause multiple toxic effects, such as generating reactive oxygen species, lipid peroxidation, protein oxidation, and DNA degradation in *E. coli* cells (Chatterjee et al. 2014). However, limited field studies have evaluated the comparative efficacy of silver and copper NPs against established non‐antibiotic therapies for postpartum clinical endometritis in dairy cows.

**TABLE 1:** Relative risks and odds ratios of selected risk factors associated with clinical endometritis in dairy cows.

Risk factor	Relative risk (95% CI)	Odds ratio (95% CI)	*p* value
BCS			
BCS <2.75(low BCS)	0.18 (0.02–1.40)	0.15 (0.18–1.30)	0.055
BCS≥2.75(normal BCS, reference)	1.23 (1.01–1.49)		
Ovarian status			
Ovary with corpus luteum	1.31 (0.79–2.15)	1.75 (0.61–4.97)	0.21
Ovary without corpus luteum	0.75 (0.43–1.30)		
Calving status			
Natural parturition	1.50 (1.06–2.11)	4.00 (1.13–14.08)	0.02
Dystocia	0.37 (0.14–0.98)		
Parity			
Primiparous	0.46 (0.17–1.24)	0.35 (0.09–1.26)	0.08
Multiparous	1.30 (0.96–1.76)		
Placental expulsion			
Normal expulsion of the placenta	1.38 (1.09–1.74)	10.12 (1.21–84.64)	0.01
Retained placenta	0.13 (0.01–0.98)		

*Note*: Reference categories were used as baseline comparisons for RR and OR calculations.

The present study aimed to compare the clinical and cytological efficacy of intrauterine silver and copper NPs with 50% dextrose and PGF2α for the treatment of postpartum clinical endometritis in dairy cows.

## 2 Materials and Methods

This prospective field study was conducted on a commercial Holstein herd in Behbahan, Khuzestan (free‐stall housing; TMR fed twice daily). A total of 210 cows were screened at 30 ± 3 Days in milk (DIM), and 60 cows diagnosed with clinical endometritis (CE) were randomly allocated into four groups (*n* = 15/group): 50% dextrose, PGF2α, silver + copper NPs (Ag + Cu NPs), and untreated controls. CE was diagnosed between 21 and 35 DIM using a vaginal discharge scoring system (0–3) per Sheldon et al., with enrolment at scores 2–3 or borderline score 1 confirmed by endometrial cytology (polymorphonuclear neutrophil [PMN] > 10%) (Sheldon et al. 2006; Kasimanickam et al. 2004). Transrectal ultrasonography supported (but did not define) the diagnosis. Vaginal mucus was sampled after perineal cleaning; cytology smears were air‐dried, methanol‐fixed, Giemsa‐stained, and evaluated in 10 fields at ×400 to estimate PMN and epithelial cell percentages. Cytological cure was defined as PMN < 10% at follow‐up.

Treatments were as follows:

Dextrose: single intrauterine infusion of 200 mL 50% dextrose via sterile transcervical pipette.
PGF2α: in cyclic cows with palpable CL, cloprostenol sodium (500 mg, IM); if anovular or with a ≥10 mm follicle, a two‐step GnRH (100 µg) then cloprostenol (day 11) protocol was used.
Ag + Cu NPs: single intrauterine infusion of 50 mL NP solution containing 3 mg Ag NPs + 3 mg Cu NPs (in 0.9% saline).

The control group received no treatment.

**FIGURE 1:**
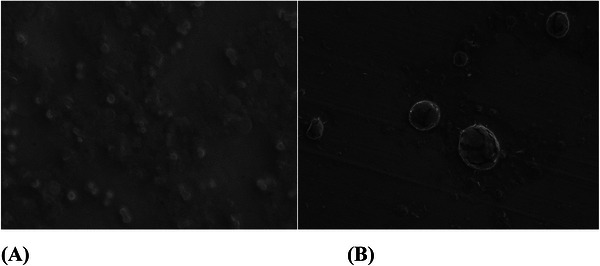
SEM morphology of Cu (A) and Ag (B) nanoparticles.

Ag NPs and Cu NPs were synthesized by chemical reduction (silver nitrate/sodium borohydride; copper sulphate/ascorbic acid in starch matrix), washed, and stored at 4°C. Mean particle sizes were ∼7 nm (Ag) and ∼6 nm (Cu) by dynamic light scattering (Quintero‐Quiroz et al. 2019). The morphology was confirmed by SEM, as shown in Figure 1.

All cows were re‐examined 14 days post‐treatment. Clinical cure was defined as the absence of purulent or mucopurulent vaginal discharge at follow‐up examination; cytological cure was PMN <10%. Reproductive indices (services per conception, days to first artificial insemination (AI), days open) were recorded from farm records. No local or systemic adverse effects were observed following treatment.

Clinical cure rates were compared by chi‐square/Fisher's exact tests. Cytology data were analyzed by one‐way analysis of variance (ANOVA) with LSD post‐hoc tests. Relative risks and odds ratios were calculated to assess associations between selected risk factors and CE occurrence. Significance was set at *p* < 0.05 (SPSS v26).

## 3 Results

**FIGURE 2:**
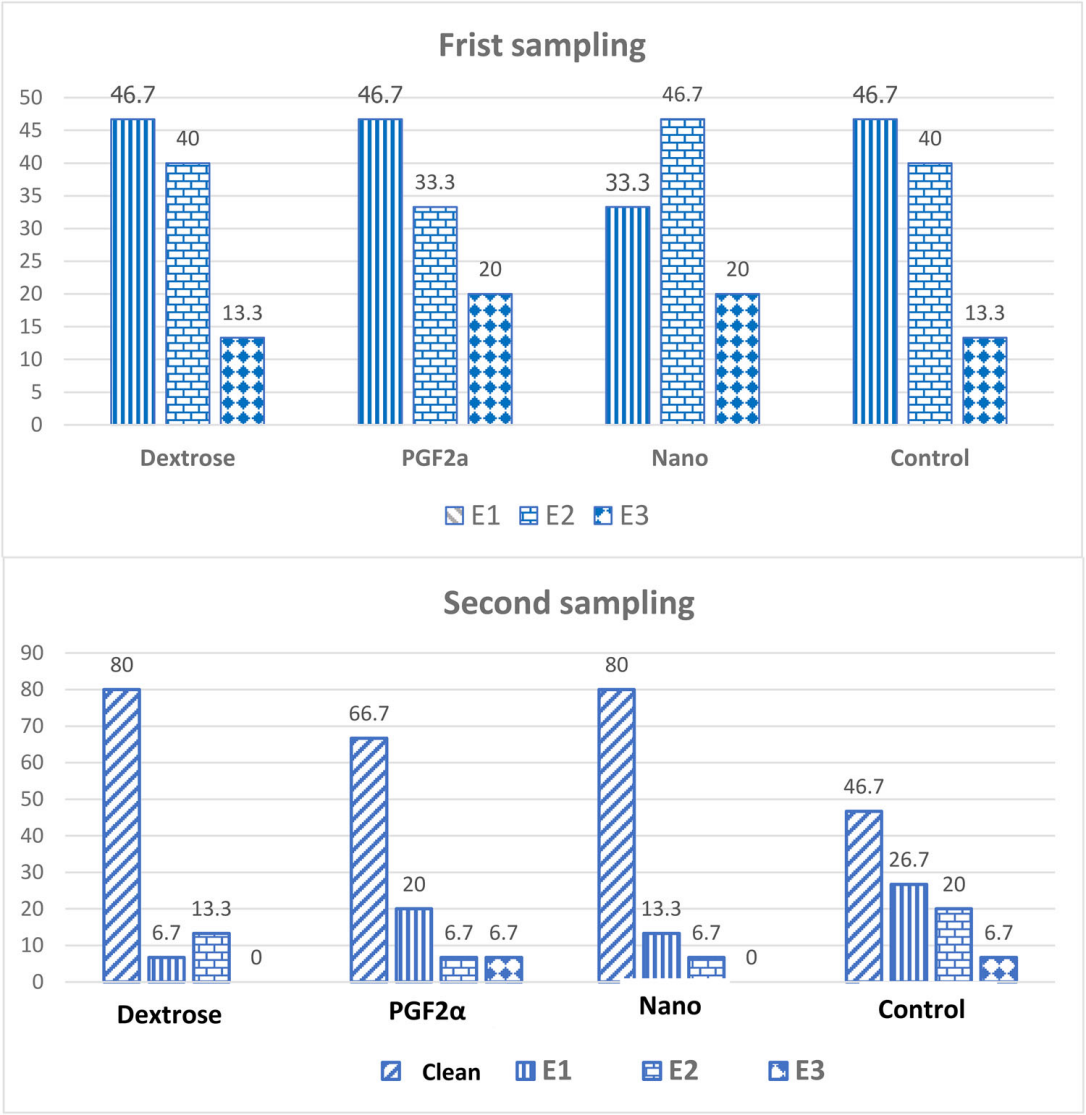
Clinical cure rate of different degrees of clinical endometritis in four treatment groups.

A total of 210 cows were screened between 21 and 35 days postpartum, and 60 animals diagnosed with CE were enrolled and evenly assigned to four treatment groups: 50% dextrose, PGF2α, silver + copper NPs (Ag + Cu NPs), and untreated controls (Table 2). The overall incidence of CE in the herd was 28.6%. Clinical cure, defined as the absence of purulent discharge and the Clinical cure, was achieved in 80% of the cows treated with either dextrose or Ag + Cu NPs, compared with 66.7% in the PGF2α group and 46.7% in untreated controls (*p* < 0.05; Figure 2). These findings indicate that both dextrose and NP therapies were significantly more effective than prostaglandin or no treatment.

**TABLE 2:** Distribution of endometritis severity across treatment groups.

		Level of endometritis			Total
		E1	E2	E3	
Treatment	Dextrose	7	6	2	15
	PGF2α	7	5	3	15
	Nano particles	5	7	3	15
	Control	7	6	2	15
Total		26	24	10	60

*Note*: The severity level of endometritis was determined based on the vaginal mucus scoring system (0–3) described by Sheldon et al. (2006). Levels E1–E3 represent increasing severity of abnormal discharge. Score 0 (absence of abnormal discharge) was not represented, as only cows diagnosed with clinical endometritis (score ≥1) were enrolled in the study. The table reflects the baseline distribution of severity across groups.

Cytological assessment demonstrated a marked reduction in PMN percentage in the dextrose (16.2 ± 14.6%) and NP (15.1 ± 18.2%) groups compared with the control group (35.7 ± 29.9%) (*p* < 0.05). The prostaglandin group (20.7 ± 25.7%) showed an intermediate response. The higher proportion of epithelial cells post‐treatment in the dextrose and NP groups further supported improved uterine epithelial recovery and reduced inflammation (Table 3).

**TABLE 3:** Mean ± SD of cytological cure rate 14 days after treatment in study groups.

Treatment groups	First sampling		Second sampling	
	Neutrophil	Epithelial	Neutrophil	Epithelial
Dextrose	54.93 ± 21.28	45.73 ± 21.13	16.20 ± 14.60a	83.13 ± 14.57a
Prostaglandin	56.60 ± 23.38	43.40 ± 23.38	20.66 ± 25.69b	79.33 ± 25.69b
Nanoparticles	55.53 ± 22.26	43.80 ± 22.93	15.06 ± 18.19a	84.93 ± 18.19a
Control	54.06 ± 20.97	81.80 ± 144.01	35.73 ± 29.95c	64.26 ± 25.95c

*Note*: Values with different letters within a column differ significantly at *p* < 0.05.

Reproductive indices improved in all treated groups relative to controls. The mean number of inseminations per conception was the lowest in the NP group (2.13 ± 1.24) and the dextrose group (2.20 ± 1.01) compared with the control (3.40 ± 1.35, *p* < 0.05). Similarly, the mean days open were shorter in treated cows (107–111 days) than in controls (155 days, *p* < 0.05). No significant difference was found between the dextrose and NP groups (Table 4).

**TABLE 4:** Mean ± SD of reproductive performance in different treatment groups.

Group of treatment	Number of AI	Days to first service	Days open
Dextrose	2.20 ± 1.01a	63.26 ± 5.27a	107.13 ± 39.01a
Prostaglandin	2.60 ± 1.35a	63.93 ± 10.34a	119.73 ± 57.64a
Nanoparticles	2.13 ± 1.24a	64.46 ± 9.53a	110.73 ± 55.19a
Control	3.40 ± 1.35c	73.26 ± 5.14b	154.86 ± 44.17b

*Note*: Values with different letters within a column differ significantly at *p* < 0.05.

The present study demonstrated that intrauterine administration of Ag + Cu NPs achieved clinical and cytological outcomes comparable to hypertonic dextrose and superior to PGF2α in cows with postpartum CE (Ahmadi et al. 2019; Makki et al. 2017). The mechanism of dextrose therapy has been attributed to its local osmotic effect, promotion of endometrial regeneration, and inhibition of bacterial growth. The comparable clinical outcomes observed with Ag + Cu NPs in this study suggest antimicrobial and possible anti‐inflammatory effects. Previous studies have shown that Ag NPs disrupt bacterial membranes and induce oxidative stress in uterine pathogens such as *Prevotella melaninogenica* and *Arcanobacterium pyogenes* (Gurunathan et al. 2018). Similarly, Cu NPs exert bactericidal activity through reactive oxygen species generation and DNA damage (Chatterjee et al. 2014).

In the current trial, the efficacy of Ag + Cu NPs was equivalent to dextrose and superior to PGF2α. This advantage is likely due to their direct bactericidal action rather than the hormonal mechanisms of PGF2α, which relies on luteolysis and secondary immune stimulation. Hussein and Hussein (2022) also reported a 70% cure rate in cows treated with intrauterine Ag NPs, supporting the reproducibility of our results.

## 4 Limitations and Practical Implications

The main limitation of this study is the modest sample size per group (*n* = 15), which may reduce statistical power for detecting minor differences. Nonetheless, the consistency of outcomes between dextrose and NP treatments supports the biological relevance of these findings. Importantly, both treatments are antibiotic‐free, thereby eliminating concerns about drug residues and bacterial resistance. In addition, long‐term safety and residue kinetics of intrauterine metallic NPs require further investigation.

## 5 Conclusion

Intrauterine administration of silver and copper NPs achieved clinical and cytological cure rates comparable to 50% dextrose and superior to prostaglandin treatment in cows with postpartum clinical endometritis. These results highlight the potential of metal NPs as effective, residue‐free, non‐antibiotic therapies to improve uterine health and reproductive performance in dairy herds.

## Author Contributions

Meysam Makki: methodology, formal analysis, and writing – original draft preparation, review, and editing. Seyed Mohammad Taha Mousavi: project administration, investigation. Saad Goorani Nejad: conceptualization. M Mohammad Khosravi: resources.

## Funding

The Shahid Chamran University of Ahvaz supported this work (grant number: scu vc1401.39117).

## Ethics Statement

All protocols complied with ARRIVE guidelines, and all procedures strictly followed the Animal Scientific Procedures Act (1986).

## Conflicts of Interest

The authors declare no conflicts of interest.

## Data Availability

The data supporting this study's findings are available from the corresponding author upon reasonable request.
